# Effect of training on navigated frameless and frame-based stereotactic brain biopsies: a retrospective comparison of staff neurosurgeon and trainee perioperative performance and complications

**DOI:** 10.1007/s00701-026-06948-7

**Published:** 2026-06-12

**Authors:** H. Joswig, S. H. Nestle, J. M. Funk, S. Sultanova, C. Doenitz, T. Seyfried, J. Augustin, F. Grassmann, U. Träger, S. Blecha

**Affiliations:** 1 HMU Health and Medical University Potsdam, Olympischer Weg 1, 14471 Potsdam, Germany; 2 Department of Neurosurgery, Ernst von Bergmann Hospital, Charlottenstraße 72, 14467 Potsdam, Germany; 3 Department of Anesthesiology, Regensburg University Hospital, University of Regensburg, Regensburg, Germany; 4 Department of Neurosurgery, Regensburg University Hospital, University of Regensburg, Regensburg, Germany; 5 Department of Anesthesiology, Vilshofen Hospital, Vilshofen, Germany; 6 Department of Epidemiology and Biostatistics, HMU Health and Medical University Potsdam, Potsdam, Germany

**Keywords:** Biopsy, Complication, Outcome, Resident training, Risk, Safety, Stereotaxy, Surgical education

## Abstract

**Background:**

Resident physicians should be integrated early into a broad spectrum of surgical procedures during postgraduate training. At the same time, medical standards must be maintained and patient safety ensured. This study investigates whether resident involvement in stereotactic brain biopsy affects perioperative performance and complications.

**Methods:**

This retrospective, two-center study compared 217 consecutive patients undergoing navigated frameless and frame-based stereotactic brain biopsies performed either by a supervised neurosurgery resident (teaching cases) or by a BCFN (non-teaching cases). The primary endpoint was the occurrence of a complication. Operation and anesthesia time as well as hospitalization length served as secondary endpoints. Categorical data were analyzed using Pearson’s chi-square and Fisher test, and continuous data using the Mann–Whitney U Test.

**Results:**

A total of 217 biopsies were stratified into *n* = 60 (27.6%) teaching cases and *n* = 157 (72.4%) non-teaching cases. Complication rates (asymptomatic hemorrhages) were comparable with 1/60 (1.7%) for resident-performed cases and 3/157 (1.9%) for BCFN cases. Mean operation time for navigated stereotactic biopsies was 38.1 ± 14.8 min for residents and 44.7 ± 22.6 min for BCFNs (*p* = 0.133). For frame-based biopsies, mean operation time performed by residents was 47.5 ± 32 min and 32.9 ± 9.9 min for BCFNs (*p* = 0.047). Anesthesia time for navigated biopsies was 119.2 ± 39.6 min for residents and 124.7 ± 27.9 min for BCFNs (*p* = 0.049). For frame-based biopsies, anesthesia time was 164.5 ± 98 min for residents and 111.8 ± 28.4 min for BCFNs (*p* < 0.01). Residents completed frameless biopsies faster than frame-based ones in terms of anesthesia time (*p* < 0.01) whereas BCFN were faster with frame-based procedures (operative time (*p* < 0.01) and anesthesia time (*p* < 0.01)). Mean hospitalization time was significantly longer in the BCFN group with 12.5 ± 15 vs. 7.52 ± 8.8 days (*p* = 0.015). There was no significant correlation between neurosurgical residents' year of training and the above parameters.

**Conclusions:**

The study supports the safety of supervised early surgical education for stereotactic brain biopsies. Residents require significantly more time for frame-based cases than BCFN, but complication rates remain comparable.

## Introduction

While simulation training in neurosurgery efficiently flattens learning curves [6], hands-on operating room training is regarded as the most valuable [20]. Previous studies have demonstrated the safety of neurosurgical resident involvement in spinal [11, 12, 21, 23, 24], and cranial procedures [10, 13, 14, 17]. However, more data are needed to confirm that patients can safely undergo surgery performed by supervised residents. This discussion has become particularly relevant following implementation of European work-hour restrictions [1, 2, 15, 19].

Brain biopsies can establish a pathological diagnosis of tumors and inflammatory conditions [18], and are considered an essential technique for a neuro-oncology expert surgeon to master [7]. Besides a burr-hole trepanation, this procedure involves perforating healthy brain and tumor tissue. Depending on the technique, the risk of intracranial hemorrhage ranges from 5.1% to 14.2% for frame-based and 2.4% to 17.8% for navigated frameless biopsies [4]. We therefore tested the null hypothesis that complication rates of procedures performed by supervised residents do not differ from those performed by experienced board-certified faculty neurosurgeons (BCFNs).

## Methods

### Study design

The electronic medical records of all brain biopsies performed between March 2018 and June 2025 at Ernst von Bergmann Hospital, Potsdam, and between July 2017 and November 2024 at Regensburg University Hospital were retrospectively reviewed. Cases involving open brain biopsy via a craniotomy, combined procedures, or missing data were excluded. Group assignment was determined by the surgeon performing the index case. Cases were thus dichotomized into teaching and non-teaching categories. Teaching cases were those (mostly) performed by a resident in postgraduate year (PGY) 1–7 under direct BCFN supervision (Level 2 per Whitfield et al. [25]). Non-teaching cases were performed by a BCFN, often with another BCFN or resident assisting.

### Operations

#### Frameless (navigation-guided) brain biopsy

Following image acquisition with contrast-enhanced MRI, the entry/target point and optimal cortical trajectory that avoids sulci and blood vessels [9] were planned using neuronavigation software (StealthStation S8, Medtronic, or Brainlab). Under general anesthesia, surface registration was carried out, and the entry point was marked. A twist drill hole was created with the burr or perforator, the dura sharply opened, the cortex coagulated and incised. The Vertek (Medtronic) or VarioGuide (Brainlab) biopsy needle system was advanced to the target along the pre-calculated trajectory under continuous navigation guidance to retrieve one or more tissue samples. Patients were monitored in the intermediate care unit, and postoperative CT was performed the following day.

#### Frame-based stereotactic brain biopsy

Preoperative imaging and target planning were completed using the same neuronavigation system. Under general anesthesia, a stereotactic frame (Riechert-Mundinger, Inomed, or Zamorano-Dujovny-System, Inomed) was affixed to the skull. Contrast-enhanced CT scans were then obtained and fused with the preoperative MRI to calculate the stereotactic coordinates, which were set at the frame. Following twist drill trepanation, dural opening, and cortical coagulation and incision, the needle was advanced to the target points and at least one biopsy taken. Postoperative management followed the same protocol as above.

#### Sample size calculation

No direct comparison of complication rates between residents and BCFNs for brain biopsies was found in the literature. A clinically relevant difference is present when the complication rate in one group is at least twice as high as in the reference group. Based on heterogeneous reports on complication frequencies for the procedures in question, complication rates between 10 and 20% were assumed. Sample size calculation indicated that *n* = 120 patients were required to detect this difference with 80% power and α = 0.05.

Previous studies on resident education had comparable case numbers *n* = 102 [23], *n* = 104 [24], *n* = 165 [14], *n* = 240 [10], *n* = 253 [17], *n* = 287 [21], *n* = 320 [13], *n* = 354 [11], and *n* = 471 [12].

#### Data collection

Baseline characteristics, including age, sex, presenting symptoms, prior brain biopsy, tumor location and volume, technical aspects of surgery, trajectory length, use of perioperative antibiotics, presence of an assistant, procedure and anesthesia duration, and hospitalization length, were recorded. Postoperative CT scans were reviewed for intracranial hemorrhage (minor hemorrhage < 5mm^2^; major hemorrhage > 5mm^2^) [18], and clinical records were screened for other complications, including infections and new neurological deficits.

#### Primary and secondary endpoints

The primary endpoint was the occurrence of intra- or postoperative complications. Secondary endpoints were operation/anesthesia time and hospitalization length (Table 1).

**Table 1:** Baseline characteristics of 217 cases undergoing a frame-based stereotactic or frameless navigation guided brain biopsy by a staff attending (*n* = 157) or supervised neurosurgery resident (*n* = 60)

		Staff (*n* = 157)	Trainees (*n* = 60)	*p*-value
Age (years)		61.9 ± 15	67.3 ± 14.5	** < 0.01 †**
Gender	Male	77 (49%)	26 (43.3%)	0.451 †
	Female	80 (51%)	34 (56.7%)	
Presenting Symptoms	Sensory deficit	13 (8.3%)	1 (1.7%)	1 ☨
	Motor deficit	73 (46.5%)	28 (46.7%)	0.982 †
	Visual problems	19 (12.1%)	8 (13.3%)	0.806 †
	Speech deficit	56 (35.7%)	21 (35%)	0.927 †
	Epileptic seizure	46 (29.3%)	12 (20%)	0.166 †
	Headache	28 (17.8%)	10 (16.7%)	0.840 †
	Personality changes	27 (17.2%)	7 (11.7%)	0.316 †
Tumor localization	Frontal	84 (53.5%)	24 (40%)	0.075 †
	Parietal	61 (38.9%)	22 (36.7%)	0.767 †
	Temporal	32 (20.4%)	21 (35%)	**0.025 †**
	Occipital	11 (7%)	8 (13.3%)	0.14 †
	Insular	3 (1.9%)	3 (5%)	0.351 ☨
	Cerebellar	4 (2.5%)	0 (0%)	0.578 ☨
	Right	59 (37.6%)	16 (26.7%)	0.303 †
	Left	78 (49.7%)	36 (60%)	
	Bilateral	20 (12.7%)	8 (13.3%)	
Diagnosis	Glioma	87 (55.4%)	33 (55%)	0.956 †
	WHO grade 1 2 3 4	1/87 (1.2%) 6/87 (6.9%) 7/87 (8%) 73/87 (83.9%)	1/33 (3%) 1/33 (3%) 2/33 (6%) 29/33 (87.9%)	-
	Lymphoma	34 (21.7%)	11 (18.3%)	0.589 †
	Metastasis	4 (2.5%)	3 (5%)	0.398 ☨
	Abscess	0 (0%)	1 (1.6%)	0.276 ☨
	Encephalitis	1 (0.6%)	2 (3.3%)	0.186 ☨
	Other	29 (18.5%)	9 (15%)	0.690 †
	Non-diagnostic	2 (1.3%)	1 (1.6%)	1 ☨
Previous brain biopsy		11 (7%)	6 (10%)	0.463 †
Surgery type	Frameless navigation-guided	86 (54.8%)	37 (61.7%)	0.36 †
	Frame-based stereotactic	71 (45.2%)	23 (38.3%)	
Tumor volume	mm^3^	39.7 ± 48.5 *n* = 29	29.5 ± 34.1 *n* = 46	0.892
Trajectory (mm)	Cortex to tumor margin	23.8 ± 15.4 *n* = 29	24.8 ± 14.7 *n* = 46	0.687
	Cortex to target point	32 ± 17.4 *n* = 29	36.9 ± 16.7 *n* = 46	0.19
Antibiotics	Cefazolin	155 (98.7%)	59 (98.3%)	1 ☨
	Clindamycin	2 (1.3%)	1 (1.7%)	
Assistant present at surgery		133 (84.7%)	60 (100%)	** < 0.01 ☨**
Operation time (min)				
…navigated biopsy …frame-based biopsy		44.7 ± 22.6 32.9 ± 9.9	38.1 ± 14.8 47.5 ± 32	0.133 † **0.047 †**
Anesthesia time (min)				
…navigated biopsy …frame-based biopsy		124.7 ± 27.9 111.8 ± 28.4	119.2 ± 39.6 164.5 ± 98	**0.049 †** ** < 0.01 †**
Subsequent brain tumor surgery after biopsy		13 (8.3%)	3 (5%)	0.565 ☨
Hospitalization time (days)		12.5 ± 15	7.52 ± 8.8	**0.015 †**

Categorical variables are presented as count and percent, nominal variables as mean and standard deviation; † Mann–Whitney U Test, Chi^2^, ☨ Fisher

#### Statistical methods

Categorical variables were compared using Pearson’s chi-square or Fisher’s exact test. Continuous variables were analyzed using the Mann–Whitney U test. To assess potential confounding by age, we performed multivariable regression analyses with complications, operation/anesthesia times, and hospitalization time as dependent variables and age as a covariate. Adjusted effect estimates are reported with 95% confidence intervals. Spearman rho correlation analysis was performed for PGY. All analyses were performed using IBM® SPSS® Statistics (version 30.0.0.0.; IBM Corp., Armonk, NY, BM, USA), except for the calculation of the risk differences in Table 2, which was performed using R version 4.3.3 with ratesci version 1.0 was used. *P*-values < 0.05 and < 0.01 were considered statistically significant.

**Table 2:** Complications in 217 frame-based stereotactic or frameless navigation guided brain biopsy cases performed by a staff attending (*n* = 157) or supervised neurosurgery resident (*n* = 60)

Complications	Staff (*n* = 157)	Trainees (*n* = 60)	*p*-value	Risk difference [95%CI]
Any	3 (1.9%)	1 (1.7%)	1	−0.1% [−4.2%; 6.1%]
Minor hemorrhage	1 (0.6%)	0 (0%)	-	not applicable
Major hemorrhage	2 (1.3%)	1 (1.7%)	1	0.6% [−3.2%; 6.7%]
Infection	0 (0%)	0 (0%)	-	not applicable

Categorical variables are presented as count and percent; *CI* Confidence Interval; ☨ Fisher

#### Ethics

All procedures performed in studies involving human participants were in accordance with the ethical standards of the Ethics Committee of the Brandenburg State Medical Association (Landesärztekammer Brandenburg) (2024–90-BO-ff) and with the 1964 Helsinki declaration and its later amendments or comparable ethical standards. The study was registered in the German Clinical Trials Register (DRKS0003476). Due to the retrospective nature of the study, informed consent was waived.

## Results

Out of 217 stereotactic brain biopsy cases, 60 (27.6%) were performed by supervised neurosurgery residents and 157 (72.4%) by BCFNs. As expected by the nature of surgical mentoring, an assistant was present in 100% of the trainee cases and during 84.7% of the staff procedures (*p* < 0.01). Patient baseline characteristics (Table 1) were well balanced except for significantly higher age in the trainee cohort (67.3 ± 14.5 years vs. 61.9 ± 15 years; *p* < 0.01) and significantly longer mean hospitalization time in the BCFN group (12.5 ± 15 vs. 7.52 ± 8.8 days; *p* = 0.015). In age-adjusted linear regression, hospitalization time was associated with older age (slope in days per year of age: 0.18, *p* < 0.01). No significant associations were found for operation/anesthesia times or complications in the adjusted models. No significant differences were observed in preoperative neurological symptoms, with motor deficits most common. Tumor localization differed significantly between groups for temporal lesions (trainees 35% vs. BCFNs 20.4%; *p* = 0.025). Previous brain biopsy (trainees 10% vs. BCFNs 7%; *p* = 0.463) and subsequent tumor surgery (5% vs. 8.3%; *p* = 0.565) showed no significant group differences. Half of final histopathological diagnosis in both groups were gliomas—predominantly grade 4 (glioblastoma); lymphomas were the second most common. In three cases (1.4%), frame-based biopsies were non-diagnostic and required repetition. In one trainee case and one BCFN case, the target was missed, yielding only peri-tumoral inflammatory tissue on histopathology. In another BCFN case, biopsy showed non-specific gliosis; repeat sampling later confirmed low-grade glioma. This translates to a diagnostic yield of 59/60 (98.3%) for trainees and 155/157 (98.7%) for BCFN.

Navigated biopsies required similar operation times in both groups, but frame-based biopsies took significantly longer for supervised trainees (47.5 ± 32 vs. 32.9 ± 9.9 min; *p* = 0.047). Anesthesia time for navigated biopsies was slightly shorter for residents than for BCFNs (119.2 ± 39.6 vs. 124.7 ± 27.9; *p* = 0.049). However, frame-based procedures were significantly longer for residents than for BCFNs (164.5 ± 98 vs. 111.8 ± 28.4 min; *p* < 0.01).

Residents completed frameless biopsies faster than frame-based ones in terms of anesthesia time (*p* < 0.01). Conversely, BCFN were faster with frame-based procedures for both, operative time (*p* < 0.01) and anesthesia time (*p* < 0.01).

Overall complication rates were low and comparable between groups (Table 2).

No significant correlation was found between neurosurgical residents' PGY level (1–7) and complications, operation/anesthesia time, or hospitalization length (Table 3).

**Table 3:** Correlation analysis between resident postgraduate year (1–7) with complications, operation time, anesthesia time and hospitalization length

Outcome parameter	Correlation coefficient	*P*-value
Complications	0.075	0.568 †
Operation time		
…navigated biopsy …frame-based biopsy	0.044 −0.019	0.795 † 0.932 †
Anesthesia time		
…navigated biopsy …frame-based biopsy	0.012 0.134	0.942 † 0.541 †
Hospitalization length	−0.139	0.284 †

† Spearman rho

## Discussion

Intracranial pathologies frequently necessitate tissue sampling for comprehensive diagnostics and therapy. Stereotactic biopsies, as a minimally invasive method, have become the standard approach, particularly for diagnosing multiple brain lesions without an identified primary tumor, and deep lesions such as those in the corpus callosum [18]. Recent studies have found that frameless navigation offers advantages over classical frame-based methods, including greater flexibility in patient positioning and shorter operation times [4].

One key aspect of operative training is the level of supervision provided to residents. Residents progress through sequential stages—from observing to participating to performing procedures independently [5]. Supervised participation of neurosurgical residents in biopsies did not adversely affect complication rates in the present cohort. This mirrors findings from other neurosurgical fields such as anterior cervical discectomy and fusion [21], cranioplasty [10], lumbar disc herniation and spinal stenosis decompression [12, 23, 24], and injections [11], burr-hole trepanation for chronic subdural hematoma [17], ventriculoperitoneal shunt implantation [13], and percutaneous glycerol rhizotomy for trigeminal neuralgia [14]. Large database analyses further support these results: Lim et al. [16] and Bydon et al. [3] reported no significant increase in morbidity or mortality associated with resident involvement across several thousand neurosurgical procedures, even after adjustment for case complexity.

Case time serves as an important surrogate marker for surgical performance. In this study, frame-based procedures performed by residents required longer operation and anesthesia times than those performed by BCFNs, consistent with previous educational series showing prolongation in teaching cases [10–12, 17, 21, 24]. Interestingly, residents as first surgeon were quicker with frameless cases than frame-based cases, while the opposite held true for BCFNs. Younger residents may have been more familiar with the interactive software than the older BCFN generation, which could have partly compensated for their lower surgical experience during frameless biopsies; however, this interpretation remains speculative and requires further study. Dhawan et al. [4] noted that frameless biopsies are associated with shorter procedure times, as time-consuming frame application and preoperative frame-based imaging are eliminated. These findings suggest that modern frameless techniques may facilitate early resident involvement in surgical training, as they appear to be easier to learn without compromising patient safety.

From a clinical perspective, reduced procedure duration benefits elderly or comorbid patients by minimizing anesthesia and immobilization time. However, Dhawan et al. [4] emphasize that this time advantage must be weighed against comparable diagnostic yield and safety. The choice between frameless and frame-based biopsy should consider local availability, team experience, and individual patient factors [4]. Educationally, this prolongation appears acceptable, as it is not associated with higher complication rates or worse outcomes and reflects time needed for teaching, feedback, and competency acquisition.

Hospitalization time is another clinically relevant endpoint for evaluating the safety of resident involvement. In this cohort, length of stay was dissimilar between teaching and non-teaching cases. Previous studies [21, 23, 24] as well as large-scale analyses have not identified resident participation as an independent predictor of prolonged hospitalization after adjustment for patient- and procedure-related risk factors [3, 16]. On the other hand, in some cohorts [13, 17], patients operated on by supervised residents stayed significantly longer, and in one study [12], this was the case for the BCFN group.

Beyond individual endpoints, the broader context of neurosurgical education in Europe and North America underscores the need to integrate residents into core procedures like stereotactic brain biopsies. European legal frameworks and national curricula aim to harmonize training standards while safeguarding patient safety [1, 19]. Comparative work on operative exposure shows that residents accumulate substantial case volumes during training, with differences between countries not translating into compromised safety under appropriate supervision [2, 15, 22]. Surveys of graduates and residency program directors confirm that high-quality patient care and readiness for independent practice depend on early, extensive operative exposure [2], including technically demanding procedures like stereotactic brain biopsies [5]. Whitfield et al. [25] expect full competency by the end of neurosurgery residency training. Haji and Steven [8] state that competency for procedures such as burr-hole trepanation can be achieved quickly under adequate supervision. Future prospective studies could further characterize burr-hole-based procedures, including stereotactic brain biopsies, by incorporating structured competency assessments (e.g. Objective Structured Assessment of Technical Skills (OSATS), Accreditation Council for Graduate Medical Education (ACGME) milestones), or learning curve analyses (e.g. Cumulative sum (CUSUM) charts).

### Strengths and limitations

The bicentric design is a key strength of this study. Limitations include retrospective data collection. Teaching vs. non-teaching classification relied on operative reports listing the first surgeon, without detailed assessment of the resident's contribution or degree of BCFN guidance. Furthermore, resident-performed biopsies in this cohort were undertaken under close, legally mandated supervision, with an assistant present in all teaching cases. Thus, the results are most applicable to training environments with comparable supervision structures. Time, complications, and hospitalization length are surrogate markers that do not fully capture overall quality of care. Given the observed low complication rates, our sample size provides only limited power to detect modest but potentially clinically meaningful differences in safety outcomes. Moreover, due to the heterogeneity of intracranial pathologies in this cohort, a standardized clinical outcome assessment was not feasible. Trainee cases involved significantly older patients than staff cases (67.3 vs. 61.9 years), which may introduce age-related confounding. However, multivariate regression analyses only revealed that older patients stayed in hospital longer, and no age-effect on perisurgical timing parameters or complications could be demonstrated. Tumor volume and trajectory data were only available for a subset of cases due to institutional data retention practices, resulting in substantial and uneven missingness between groups that may introduce bias. Both frameless and frame-based procedures were performed using standard systems from Medtronic, Brainlab, Inomed according to local availability and surgeon preference. Although the sponsor had no role in data collection, analysis, or reporting, the association with a navigation system manufacturer could consciously or unconsciously influence technique selection, case mix, or interpretation. Readers should consider this when generalizing findings to settings without such relationships.

## Conclusion

The present study adds to the literature on supervised resident participation by demonstrating the safety of trainee-performed stereotactic brain biopsies. Although operation and anesthesia times were longer in teaching cases, structured operative training maintains high-quality care and prepares future neurosurgeons for independent practice without additional risk, provided appropriate supervision and institutional standards are maintained. Prior frame assembly practice outside the operating room could mitigate prolonged procedure times.

## Data Availability

No datasets were generated or analysed during the current study.

## Abbreviations

BCFN: Board-certified faculty neurosurgeon
PGY: Post-graduate year

## Declarations

### AI statement

Sections of this manuscript were edited for grammar using Perplexity Pro.
